# The Impact of the Dobbs v. Jackson Supreme Court Decision on Orthopaedic Residency Programs: A Collaborative Orthopaedic Education Research Group (COERG) Survey

**DOI:** 10.7759/cureus.67400

**Published:** 2024-08-21

**Authors:** Lucas Bartlett, Peter B White, Selina Poon, Antonia F Chen, Julius K Oni, Brent A Ponce, Randy Cohn

**Affiliations:** 1 Orthopaedic Surgery, Northwell Health, Huntington, USA; 2 Orthopaedic Surgery, Shriner's Hospitals for Children, Los Angeles, USA; 3 Department of Orthopaedic Surgery, Brigham and Women's Hospital, Boston, USA; 4 Department of Orthopaedic Surgery, John Hopkins Bayview Medical Center, Baltimore, USA; 5 Orthopaedic Surgery, Hughston Clinic, Columbus, USA

**Keywords:** policy, acgme, orthopedic surgery, abortion care, dobbs v. jackson

## Abstract

Background: Recent changes in reproductive health care policy have now led to state-specific differences in abortion care access across the United States. Members of the medical community in particular have issued concerns regarding these new policies and their potential impact on graduate medical training.

Objectives: The purpose of this study was to sample orthopaedic surgery residency programs to gauge their perceptions of the Dobbs decision and its impact on residency training.

Materials and methods: A 25-item questionnaire was developed to assess the attitudes of orthopaedic surgery residency programs on the Dobbs v. Jackson Women’s Health Organization decision. Our survey-based study was first endorsed by and then distributed amongst members of the Collaborative Orthopaedic Education Research Group (COERG). A total of 24 representatives from 24 Accreditation Council for Graduate Medical Education (ACGME) accredited orthopaedic surgery residency programs agreed to participate in the study.

Results: Twenty-four of 24 program correspondents completed the survey (100%). Of the 15 programs (68.2%) who reported that their institution does not have a contingency plan in place, only five (33.3%) see a need for one. Eighteen programs (75.0%) agreed that the ACGME should have a policy protecting residents or significant others needing reproductive care. Ten (41.7%) respondents indicated that the Dobbs decision will impact how students rank residency programs; however, none (0%) believe it will impact their ability to attract a diverse applicant pool.

Conclusion: Although some programs surveyed have a contingency plan in place, the majority believe the ACGME should develop a policy that addresses the reproductive needs of residents. Given the higher rate of pregnancy complications experienced by women training in orthopaedic surgery it is paramount to have policies that protect residents seeking reproductive care.

## Introduction

Following the United States Supreme Court Roe v. Wade decision in 1973, access to abortion care has been protected under federal mandate [1]. However, amidst growing state restrictions and “trigger laws”, this landmark precedent was effectively overturned on June 24th, 2022 with the Supreme Court’s decision on Dobbs v. Jackson Women’s Health Organization [2,3]. By dissolving federal regulations on abortion care and displacing them under individual state law, this decision dramatically affected reproductive health care across the U.S. Subsequently, over half of the states in this country currently prohibit or restrict rights to abortion care [3,4]. 

In the ensuing months, several institutions in the medical community publicized their opposition to the Dobbs decision and its potential ramifications for medical training [5-8]. In particular, the obstetrics and gynecology (Ob/Gyn) community has raised concerns over the decision's impact on graduate medical education and resident competency in reproductive care [7,8]. In response, the Accreditation Council for Graduate Medical Education (ACGME) issued a revised set of Program Requirements for Ob/Gyn residencies [9]. However, these ultimately lack an immediate solution for residents training in states with variable access to reproductive care services [8,9]. 

Although the longitudinal impact of this evolving healthcare reality remains unclear, it is apparent that the ACGME will need to adjust its policies as any program it accredits may potentially be impacted. For orthopaedic surgery residency programs, the growing number of female trainees observed in recent years increases the likelihood for issues created by the Dobbs decision [10]. A number of studies have demonstrated that female surgical residents are more likely to experience major pregnancy complications, a higher incidence of infertility, and miscarriage rates between 11-28% [11-15]. The well-being of male surgical residents, who are more likely than women to have children during residency, may also be directly affected by such restrictions if their partners require care [15,16]. As such, limiting access to reproductive care may be detrimental for both men and women physicians seeking these health services during residency training. Currently, the ACGME does not list any policies that protect residents requiring a leave of absence for reproductive health care. 

The purpose of this study was to sample orthopaedic surgery residency programs to gauge perceptions regarding the Supreme Court decision on Dobbs v. Jackson Women’s Health Organization and how this decision may impact residency applicants.

## Materials and methods

**Figure 1:**
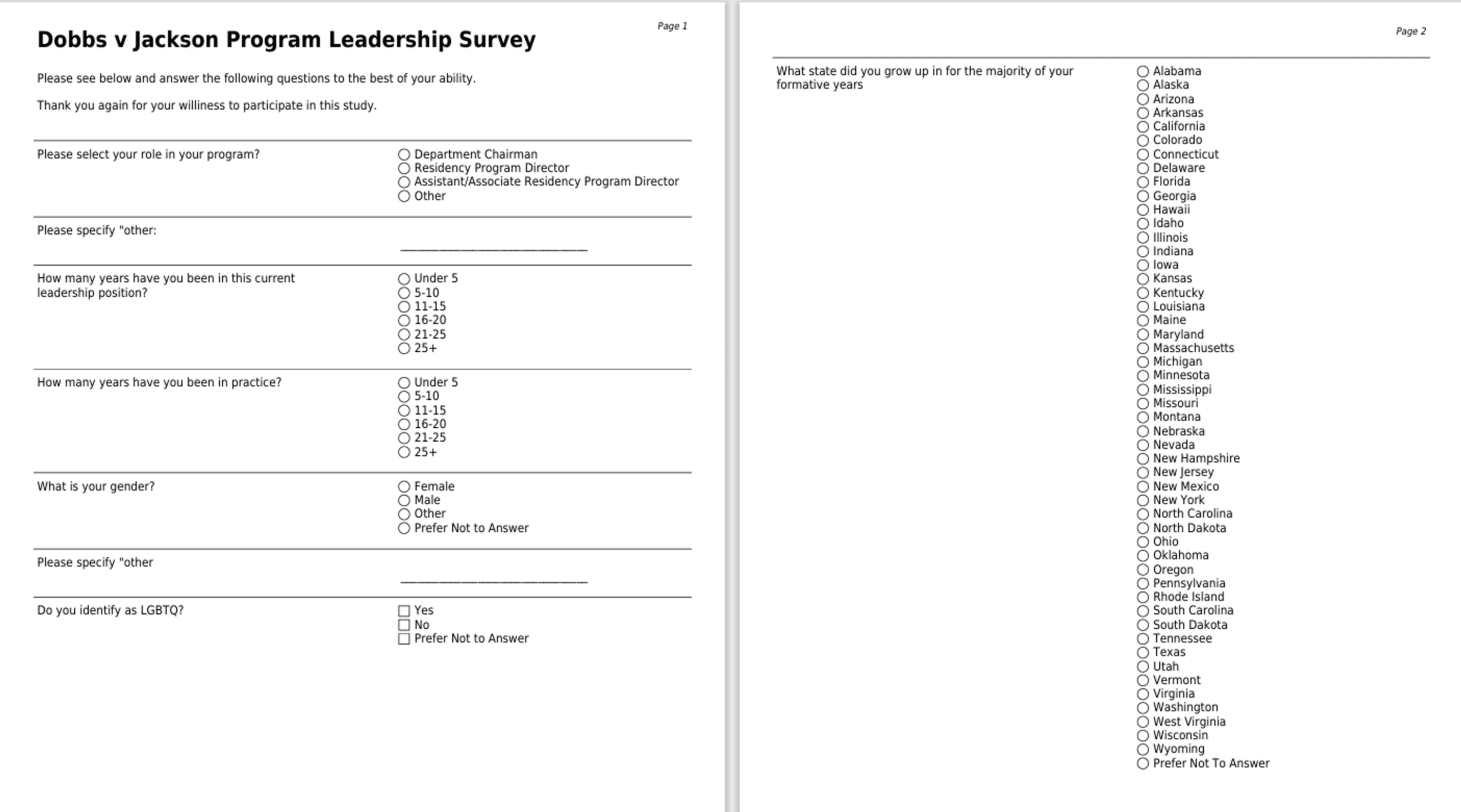
questionnaire pages 1, 2

**Figure 2:**
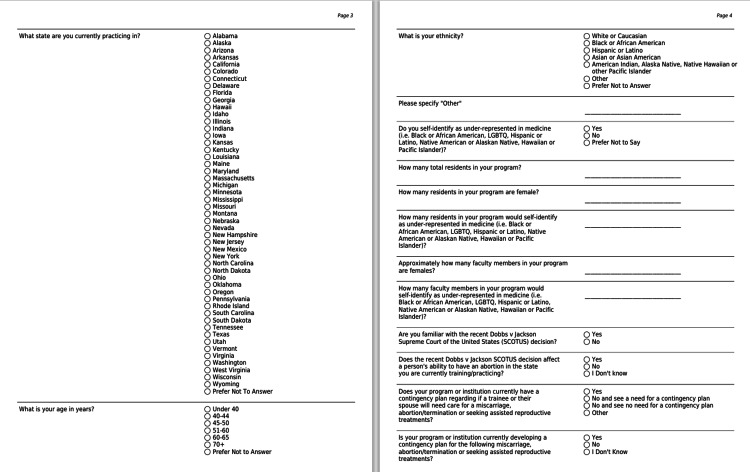
questionnaire pages 3,4

**Figure 3:**
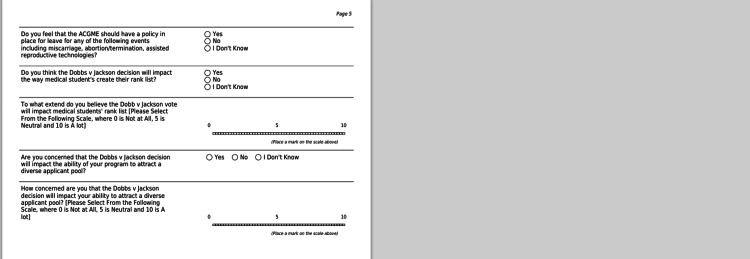
questionnaire page 5

A 25-item, mixed-response questionnaire (Figures 1-3) was developed to assess the attitudes of orthopaedic surgery residency programs on the Dobbs v. Jackson Women’s Health Organization decision. Specifically, we sought to understand how programs gauged the potential impact of this ruling on their trainees, overall program, and future residency applicants. 

After receiving Institutional Review Board (IRB) approval (ID, 22-0625-LIJ-VS) from Northwell Health Feinstein Institute for Medical Research, we presented the study protocol to the Collaborative Orthopaedic Education Research Group (COERG) board (11 members). The COERG was founded in 2021 to assist the orthopaedic training experience of resident physicians, improve the quality of faculty education, and foster professional development-related research [17]. After endorsing the project, the study protocol was presented to the entire COERG group, which includes over 100 program directors from ACGME-accredited orthopaedic residencies across the United States. A total of 24 COERG representatives agreed to participate in this study and respond in accordance with a protocol approved by the IRB [18]. 

This optional and anonymous survey was electronically distributed to correspondents from 24 orthopaedic surgery programs via email. Consent was obtained electronically and respondents completed the survey by accessing the secure hyperlink included. Follow-up emails were sent approximately one month after their initial distribution. This survey was created and distributed via the Research Electronic Data Capture software (REDCap; Vanderbilt University, Nashville, TN, USA). Upon completion, survey responses were compiled and analyzed. Both respondent and program sociodemographic factors were obtained. Descriptive statistics were used to report demographic information and all other responses.

## Results

Responder demographics 

Twenty-four of 24 program respondents completed the survey (100%). Seven respondents were female (29.2%) and 17 were male (70.8%) (Table 1). All respondents were of either Caucasian (22, 91.7%) or Asian descent (2, 8.3%). One responder identified as lesbian, gay, bisexual, transgender, or queer (LGBTQ) and underrepresented in medicine. Roles were defined as chairperson (2), program director (11), and assistant program director (4), while seven respondents did not specify their role. All unspecified respondents were COERG representatives who held core faculty roles within their respective residency programs. With regards to regional distribution, nine programs were located in the South (Georgia: 3, Alabama: 2, South Carolina: 1, Louisiana: 1, Texas: 1, Maryland: 1) seven in the Northeast (New York: 4, Pennsylvania: 1; Vermont: 1, Rhode Island: 1), two each in the Midwest (Illinois: 2) and West (California: 1, Oregon: 1), while four did not specify. Approximately 86% (18/21) of respondents currently practice in the same region where they spent their formative years. 

**Table 1:** Responder Demographics

Parameter	Responders (%)
Age (years)	
Under 40	6 (24%)
40-44	9 (37.5%)
45-50	2 (8.3%)
51-60	5 (20.8%)
Over 60	2 (8.3%)
Sex	
Male	17 (70.8%)
Female	7 (29.2%)
Ethnicity	
Caucasian/White	22 (91.7%)
Asian or Asian American	2 (8.3%)
Region of Practice	
Northeast	7 (35%)
Midwest	2 (10%)
South	9 (45%)
West	2 (10%)
Leadership Role	
Chairperson	2 (8.3%)
Program Director	11 (45.8%)
Assistant Program Director	4 (16.6%)
Unspecified	7 (29.2%)

Dobbs v. Jackson 

Twenty-one respondents (87.5%) stated they were familiar with the Dobbs decision. Five (16.7%) programs were located in a state that prohibits abortion, 14 (58.4%) with state laws protecting abortion care, and five (20.8%) were unsure of the impact in their state. A total of 22 programs reported on their contingency plans for residents or significant others needing abortion care (Table 2). Seven programs (31.8%) reported that their institution has a contingency plan. Ten programs (45.5%) reported that they do not have a contingency plan and do not see a need for one, while five programs reported that they do not have a plan but did see a need for one (22.7%). The majority of program leadership (75.0%) stated that the ACGME should have a policy protecting residents who require leave for abortion/miscarriage-related health care. Ten (41.7%) respondents indicated that the Supreme Court decision will impact how students rank residency programs in the match; however, none (0%) of the respondents believe that this will impact the ability of their program to attract a diverse applicant pool.

**Table 2:** Orthopaedic Surgery Program Perceptions on the Dobbs Decision and Impact on Graduate Medical Education ACGME: Accreditation Council for Graduate Medical Education

Parameter	Responders (%)
Does the Dobbs decision currently affect abortion rights in your state of practice?	
Yes	5 (20.8%)
No	14 (58.4%)
Unsure	5 (20.8%)
Does your program or institution have a contingency plan if trainees or their spouses require care for miscarriage, abortion/termination, or assisted reproductive treatment?	
Yes	7 (31.8)
No and don’t see a need	10 (45.5%)
No and do see a need	5 (22.7%)
Unsure	0 (0%)
Is your program or institution currently developing a contingency plan for the following: miscarriage, abortion/termination, or assisted reproductive treatment?	
Yes	1 (4.1%)
No	13 (54.2%)
Unsure	10 (41.7%)
Do you feel the ACGME should have a policy in place for residents requiring care for miscarriage, abortion/termination, or assisted reproductive treatment?	
Yes	18 (75%)
No	4 (16.6%)
Unsure	2 (8.4%)
Do you think the Dobbs decision will impact the way medical students create their rank list?	
Yes	10 (41.7%)
No	8 (33.3%)
Unsure	6 (25%)
Do you think the Dobbs decision will impact your program’s ability to attract a diverse applicant pool?	
Yes	0 (0%)
No	24 (100%)
Unsure	0 (0%)

## Discussion

**Figure 4:**
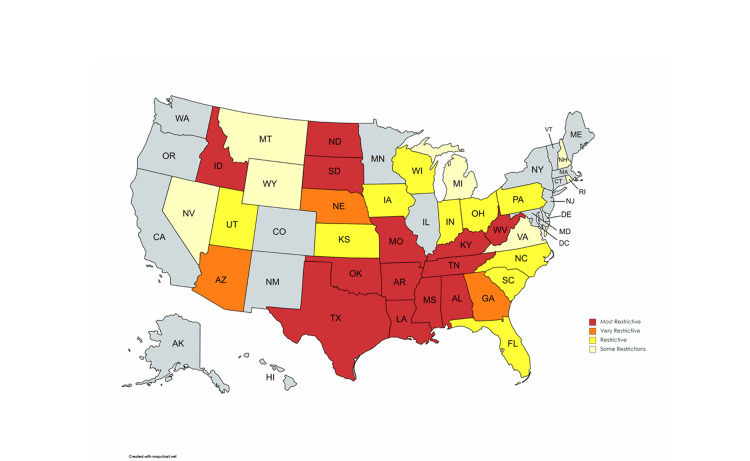
The National Distribution of States with Restricted Access to Abortion Care Adopted from the Guttmacher Institute [3]. The map reflects state policies in effect as of June 6, 2023.

In the post-Dobbs era, 26 states have total or near-total bans on abortion (Figure 4) [4]. While several studies have outlined its potential impact on health care, few have explored the implications of the Dobbs decision on resident physicians and graduate medical education [19-21]. The purpose of this study was to sample the leadership of orthopaedic surgery residency programs to gauge their perceptions regarding the Dobbs decision and how it may impact orthopaedic surgery residency programs. 

Numerous studies have demonstrated the pregnancy-related risks for female surgical residents [11-15]. A recent systematic review determined women in this setting experience a significantly higher rate of major pregnancy complications (30% v 5-15%), miscarriage (26% v 4-13%), unplanned cesarean section (25.5% v 12.5%), and infertility (33% v 10.9%) compared with the general population. Surgical residents, who often delay childbearing, were also more likely to use assisted reproductive treatment (ART) (18-28% v 5-12%) compared with non surgeon cohorts [15]. Similar rates of perinatal complications and use of ART have been identified for women training in orthopaedic surgery [22]. According to the American College of Obstetrics and Gynecology (ACOG), illness during pregnancy is one of many factors that influence a woman’s decision to terminate a pregnancy; and in cases with severe complications may be the only measure available to preserve a woman’s life [23]. However, considering the negative perceptions associated with pregnancy during training, it has been reported that less than a third of surgical residents experiencing pregnancy loss inform their employers while less than 10% take time off during or immediately after miscarriage [12,15,22,24-26]. For women who become pregnant during residency training, planned or unplanned, it’s evident that having access to reproductive care services may be critical for their health and well-being. 

Despite being one of the least diverse medical specialties, the expanding recognition of gender inequality has facilitated an increasing number of women entering the field of orthopaedic surgery [10,27,28]. With a large number of residency programs residing in states with restricted abortion care, it’s important to consider how the Dobbs decision may impact the ability of these programs to attract diversified applicant pools in future match cycles. However, only 41.7% of respondents from our study stated that the Dobbs decision will impact how medical students rank residency programs and none indicated it would impact their ability to attract diverse applicant pools. Previous literature has shown that maternity leave policies and the number of female faculty or residents are some of the least influential factors considered by women applicants in their ranking of orthopaedic surgery programs [29]. However, in the post-Dobbs era, large survey studies have indicated that nearly two-thirds of medical student respondents will preferentially apply to residency programs in states with preserved abortion care [30]. A disproportionately lower number of unique residency applicants was observed in states with abortion bans between 2022 and 2023 [31]. These reports are distinct from the perceptions of respondents in our study, which may be more reflective of regional beliefs or attitudes. Regardless, in this unfolding era of graduate medical education, program leaders of orthopaedic surgery residencies may ultimately need to consider adopting more targeted recruitment strategies to safeguard the specialty’s expanding diversity. 

Within our study, contingency plans were defined as program-level policies that support trainees in situations where current ACGME policies fail to provide adequate guidance. For example, if the non-surgeon partner of a male resident physician requires a pregnancy termination in a state with complete abortion bans, there are no ACGME policies in place to safeguard leave of absence for the time off potentially needed for out-of-state travel. Of the survey samples collected, approximately 54.6% of programs either have a contingency plan in place or see a need for one to assist trainees experiencing miscarriage/termination or those seeking ARTs. Of those programs, approximately 78% are located in states that restrict abortion. For general surgery programs, changes in department policy such as scheduling flexibility, optional research blocks, reduced on-call or operative commitments, and paid leave have been recommended as potential options to accommodate pregnant trainees [15]. Residency programs looking to create contingency plans may adopt similar strategies to permit an appropriate length of time away from work duties to seek abortion or reproductive care and treatment. Ideally, these should expand to include family leave policies as male or female partner trainees can also be affected in such settings. Additionally, given the considerable psychological impact associated with perinatal loss, residency programs should also allot time for trainees to seek grief counseling services [32,33]. 

Regardless of their perspectives on contingency plans, almost all program leaders (75%) stated the ACGME should have a policy in place protecting trainees requiring access to such care. While the ACGME and American Board of Orthopaedic Surgery (ABOS) have recent guidelines which permit up to six weeks of parental leave for orthopaedic surgery residents, no such accommodations exist for residents or partners needing prepartum or reproductive care services [34,35]. Currently, the ABOS requires 46 weeks of education per year of residency; however this can be averaged over five years, permitting trainees to take longer periods of family leave in a given year if needed [35]. While guidelines such as this provide programs with certain scheduling flexibility, their adequacy for addressing the entirety of issues that may arise from the Dobbs decision remains under speculation. Without targeted policies in place, medical training in the post-Dobbs era should be critically viewed by the ACGME to secure the well-being of resident physicians.

We report several limitations within our study. First, our survey was only distributed to COERG representatives interested in this topic, which introduces voluntary response bias. Second, considering the geographical distribution of our respondents, it’s possible our results were influenced by regional beliefs or attitudes. For example, approximately 57% of respondents practice in states without restrictions on abortion care while the majority of remaining programs are located in states with complete bans. Additionally, only one representative from each program completed the survey and their views may not be reflective of the entire program or other faculty. Furthermore, the role for seven respondents could not be confirmed. Lastly, only 24 programs were represented in total, which constitutes a small portion (12%) of ACGME-accredited programs in the United States. While we acknowledge our study represents a focal population, thus limiting its generalizability, our authors feel it brings an important conversation to light. 

## Conclusions

Most orthopaedic surgery residency programs surveyed do not have a contingency plan in place for residents or significant others needing reproductive care; however, the majority of programs surveyed believe the ACGME should have a policy protecting these residents. While some programs feel the Dobbs decision will impact how medical students prepare their rank lists, no programs believe it will impact their ability to recruit a diverse pool of applicants. As orthopaedic surgery residency programs continue to calibrate in the post-Dobbs era, the ACGME should critically monitor its effect on graduate medical education.
